# Building and Sustaining a Community Advisory Board of African American Older Adults for Volunteer Research

**DOI:** 10.1093/geroni/igaa057.3096

**Published:** 2020-12-16

**Authors:** Jamie Mitchell, Tam Perry, Vicki Johnson-Lawrence, Vanessa Rorai

**Affiliations:** 1 University of Michigan, Ann Arbor, Michigan, United States; 2 Wayne State University, Detroit, Michigan, United States; 3 Michigan State University-Flint, Flint, Michigan, United States; 4 Healthier Black Elders Center, Detroit, Michigan, United States

## Abstract

Older African Americans’ (AA) participation in health-related research is severely limited; they are not involved in sufficient numbers and for sufficient duration to ensure the applicability of advancements in medical and behavioral health. This research participation gap exacerbates older AAs vulnerability to poor health outcomes and disparities. The Michigan Center for Urban African American Aging Research employs a progressive community-based participatory model that utilizes a structured community advisory board (CAB) of older AAs in metro Detroit to oversee the research recruitment and retention of fellow AA older adult research participants. CAB members are provided ongoing training on social and behavioral health research, supported in acting as a consultancy to outside researchers where they can be compensated for their expertise and engagement, and empowered as gatekeepers of a participant research registry of over 1000 AA older adults. This model has broad potential for advancing community engaged research with AA older adults.

